# Street trees reduce the negative effects of urbanization on birds

**DOI:** 10.1371/journal.pone.0174484

**Published:** 2017-03-23

**Authors:** João Carlos de Castro Pena, Felipe Martello, Milton Cezar Ribeiro, Richard A. Armitage, Robert J. Young, Marcos Rodrigues

**Affiliations:** 1 Programa de Pós-Graduação em Ecologia Conservação e Manejo de Vida Silvestre, Instituto de Ciências Biológicas, Universidade Federal de Minas Gerais, Belo Horizonte, Minas Gerais, Brazil; 2 Laboratório de Ornitologia, Departamento de Zoologia, Instituto de Ciências Biológicas, Universidade Federal de Minas Gerais, Belo Horizonte, Minas Gerais, Brazil; 3 Spatial Ecology and Conservation Lab (LEEC), Department of Ecology, Institute of Biosciences, Universidade Estadual Paulista Julio de Mesquita Filho, UNESP, Rio Claro, São Paulo, Brazil; 4 School of Environmental and Life Sciences, Peel Building, University of Salford Manchester, United Kingdom; University of Sydney, AUSTRALIA

## Abstract

The effects of streets on biodiversity is an important aspect of urban ecology, but it has been neglected worldwide. Several vegetation attributes (e.g. street tree density and diversity) have important effects on biodiversity and ecological processes. In this study, we evaluated the influences of urban vegetation—represented by characteristics of street trees (canopy size, proportion of native tree species and tree species richness)—and characteristics of the landscape (distance to parks and vegetation quantity), and human impacts (human population size and exposure to noise) on taxonomic data and functional diversity indices of the bird community inhabiting streets. The study area was the southern region of Belo Horizonte (Minas Gerais, Brazil), a largely urbanized city in the understudied Neotropical region. Bird data were collected on 60 point count locations distributed across the streets of the landscape. We used a series of competing GLM models (using Akaike's information criterion for small sample sizes) to assess the relative contribution of the different sets of variables to explain the observed patterns. Seventy-three bird species were observed exploiting the streets: native species were the most abundant and frequent throughout this landscape. The bird community's functional richness and Rao's Quadratic Entropy presented values lower than 0.5. Therefore, this landscape was favoring few functional traits. Exposure to noise was the most limiting factor for this bird community. However, the average size of arboreal patches and, especially the characteristics of street trees, were able to reduce the negative effects of noise on the bird community. These results show the importance of adequately planning the urban afforestation process: increasing tree species richness, preserving large trees and planting more native trees species in the streets are management practices that will increase bird species richness, abundance and community functional aspects and consequently improve human wellbeing and quality of life.

## Introduction

The expansion of urban landscapes is happening at an accelerated rate. By the year 2050, two-thirds of the human population will live in cities [1], and about 60% of all the infrastructure intended to improve cities by 2030 has yet to be built [2]. The replacement of natural habitats by artificial elements—such as houses, buildings and streets—leads to disturbances and negative impacts on different biological taxa. To persist within cities, organisms need to adapt to the direct and indirect effects of environmental changes such as habitat loss and fragmentation, destruction of freshwater resources and introduction of exotic species [3]. Therefore, it is necessary to understand how these environmental changes affect the biodiversity and ecological processes essential for maintaining human quality of life and the functioning of urban ecosystems.

Birds are a highly diverse taxon and are sensitive to environmental changes in anthropogenic landscapes [4], wherein some species are more capable than others to occupy urban landscapes [5–7]. Characteristics of the urban vegetation, such as street trees, gardens and natural habitat patches, are important for the maintenance of bird populations in cities [8]. Actions such as planting native tree species [9], planning an ecological network connecting habitat patches [10] and ensuring the availability of resources for native fauna [11] increase bird species richness, abundance and diversity as well as reducing the negative effects of the urbanization process, such as biotic homogenization [12].

Despite the considerable amount of information about the effects of urban green elements, the urban matrix [7], and roads on birds [13], little is known about how birds are influenced by disturbances and vegetation characteristics of streets. Traffic volume and the size of the vegetation gap affects the movement of songbirds [14] and traffic noise has an influence on antipredator behavior [15], causing changes in song patterns [16]. However, when the urban vegetation is properly managed, streets need not be completely negative to urban birds. Species can use street trees to move between urban parks and habitat patches [17]. Streetscapes that contain predominantly native tree species, increase native bird species richness and abundance, and the bird community is more similar to that in natural habitat patches than in streetscapes, which are composed mainly of exotic tree species [18].

Since different bird species can use urban vegetation to different degrees [8], such species differences must be taken into consideration when evaluating how urbanization affects bird communities. In recent decades, functional diversity approaches have been widely used to understand the influences of human activities on biodiversity. In general, this can be defined as the range and the value of functional traits (such as body mass and foraging substrate) of a determined community that influence ecosystem functioning [19], thus incorporating the differences between species. Indices derived from this approach have the potential to reveal the processes that shape communities, and can help in understanding how biodiversity interacts with environmental constraints [20]. The use of different indices has been considered a better strategy to assess all the functional aspects of a community, rather than try to represent them in a single value, such as functional diversity *per se* [21,22]. Thus, the union between these indices and taxonomic information about the community makes it possible to identify groups of organisms that are sensitive to anthropogenic disturbances, as well as organisms that are able to live and exploit human-dominated landscapes.

However, knowledge about the effects of urbanization on birds is geographically biased—although Neotropical cities are undergoing one of the most rapid and intense urbanization processes [1], they remain understudied [23]. Following the global trend, Neotropical cities are concentrated within and near highly productive areas such as coastal zones and major riverine systems [3]. Although Neotropical cities are highly urbanized, they suffer from some of the world’s largest social and economic inequalities, which pose a threat to several biodiversity hotspots [24].

**Fig 1:**
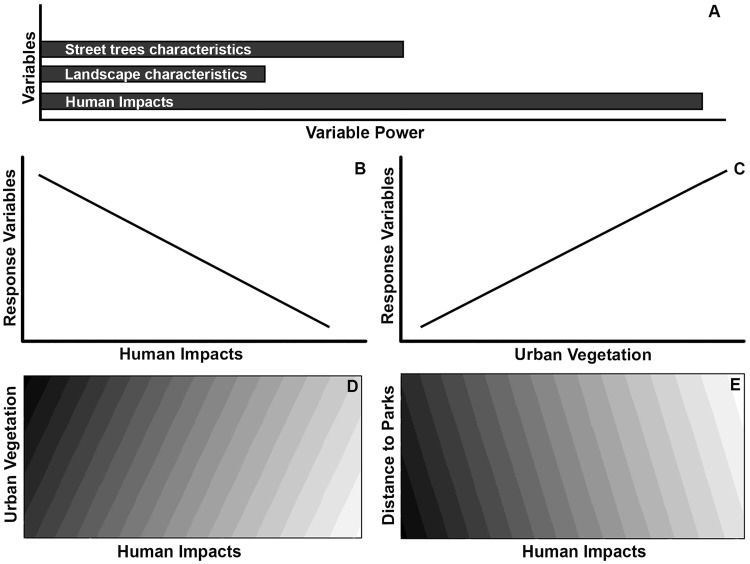
Hypothesis of the effects of the urban vegetation (street trees and landscape characteristics) and human related variables on the urban bird community inhabiting streets. In Figures 1C and 1D, darker colors represent higher response variables values (taxonomic data and functional diversity indices).

Considering the importance of understanding biodiversity in urban ecosystems, particularly on streets, this study aimed to assess how human impacts and urban vegetation—represented by characteristics of street trees (canopy size, tree species richness and the proportion native tree species) and characteristics of the landscape (amount of vegetation and distance to parks)–influence bird species inhabiting the streets of a largely urbanized Neotropical city. We hypothesized that the negative effects of urbanization on birds [7] from human impacts will have the strongest effect on taxonomic data and functional diversity indices of the urban bird community, followed by the characteristics of street trees (we expected this patterns since birds are highly influenced by the availability of resources—such as food and nesting places [25]) and then the characteristics of the landscape (Fig 1A). We also hypothesized that variables related to human impacts and the distance to parks will have negative influences on the taxonomic data and functional diversity indices (Fig 1B), while the remaining characteristics of urban vegetation will have positive influences (Fig 1C). Finally, we hypothesized that when urban vegetation and human impacts are considered in the same model, the former will influence positively the taxonomic data and functional diversity indices, despite the strongest negative effect of the human impacts (Fig 1D). However, we expected that the distance to parks will negatively affect the urban bird community (Fig 1E).

## Material and methods

### Study area

**Fig 2:**
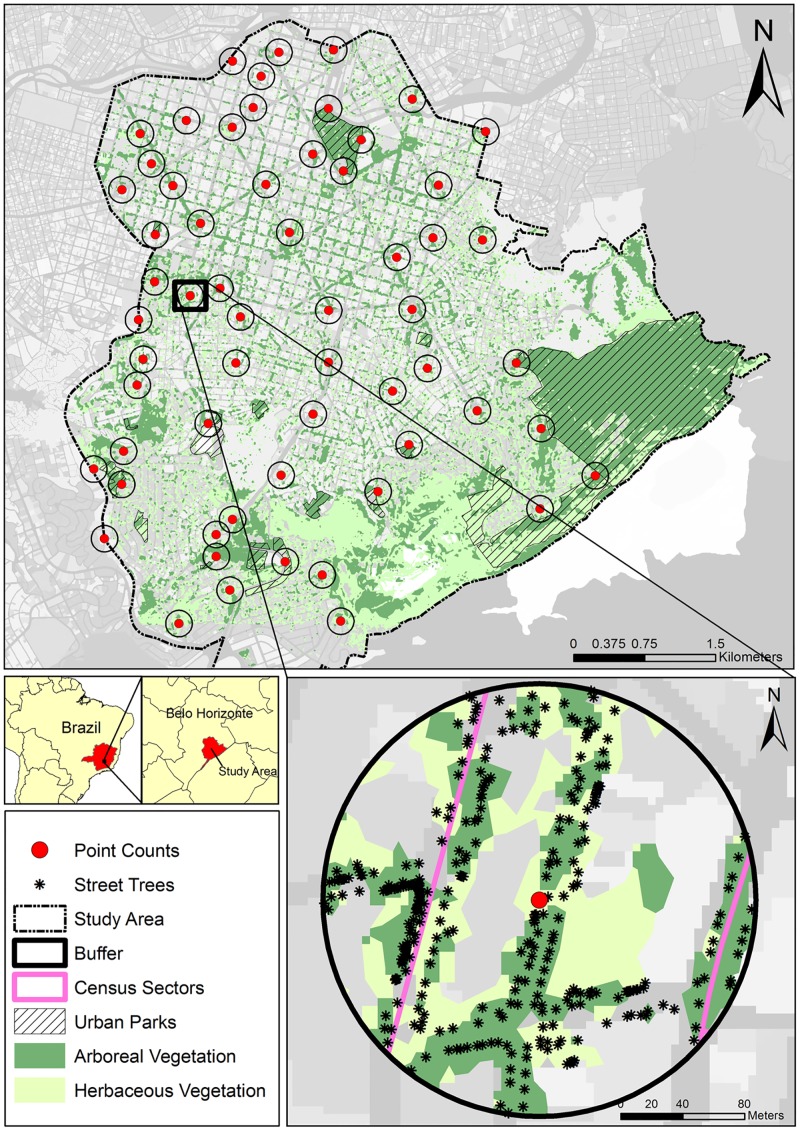
Southern region of Belo Horizonte (Minas Gerais, Brazil), with the point count locations where birds were observed on the streets of the study area. The circles represent 140m radius buffers around each sampling point. Urban vegetation elements and census sectors are highlighted within one of the 140m radius buffers. Arboreal and herbaceous vegetation data was obtained from Pena et al. [26].

The study area was the southern region of Belo Horizonte city (W 19° 55' 37", S 43° 56' 34") one of the first planned cities in Brazil and the Minas Gerais state capital (Fig 2). The southern region of Belo Horizonte covers 31.7 km^2^, which includes the oldest part of the city (est. 1897), an area of approximately 9 km^2^. According to the land use/land cover map developed by Pena et al. [26], almost half of the study area is occupied by arboreal and herbaceous vegetation, concentrated at the southern portion of the study area within urban parks (Fig 2). The rest of the landscape is composed of scattered green areas and public squares, and street trees composed mainly of exotic species [26]. In our study area, it is possible to find Cerrado and Atlantic Forest remnants, as well as *campos rupestres* (rocky fields) and *campos de altitude* (high altitude fields) patches, typical mountain grasslands, located in the higher portions of the city (1300 to 1400 meters a.s.l.), which are located in the southern part of our study area (Fig 2).

### Point counts selection and bird community data

We selected 60 point count locations [27] distributed in streets through the southern region of Belo Horizonte. Point selection aimed to represent the variation of the influences of the streets and arboreal and herbaceous vegetation within the study area. We sampled points that, within a 50 meters radius, have large amounts of arboreal and herbaceous vegetation but low street density, as well as points within the city center, with high street density, and small amount of herbaceous or arboreal vegetation (S1 Text, S1 Fig). Point counts were at least 200 meters away from each other.

We conducted a pilot study to define the number of visits and the duration of the point counts, and we observed that three 20-minute visits were sufficient to obtain information about bird species richness in the streets of the study area. Fieldwork started 30 minutes after sunrise and extended during the first three hours of daylight on days with favorable weather (sunny and non-windy days). To define the point sampling order, the first point count was randomly selected. After 20 minutes of sampling, the observer walked to the next nearest sample point. This process was repeated during the first three hours of the day. Point counts were conducted only on working days to avoid great variation in people and vehicles in circulation.

Point counts were conducted by one trained ornithologist (J.C.C. Pena) between September 2014 and January 2015. This period coincides with birds’ breeding season, during which different migratory species visit Belo Horizonte [28,29]. All bird species (breeding and non-breeding birds) within a 50 m radius around the center of the point count were recorded visually or acoustically and counted. Monospecific flocks were considered to be up to a number of 20 individuals. The following behaviors were recorded: flying, resting or performing activities (e.g.: feeding, nesting). Birds suspected of being captive or pets were not included.

To analyze the effects of human impacts and urban vegetation on the urban bird community, we only considered species and individuals registered resting, feeding or nesting in the point count locations. Therefore, we excluded from our analysis species and individuals that were recorded only flying—they could be only moving through the urban landscape—and singing—they could be within an urban park or habitat patch nearby the point count. Thus, our analysis was limited to species that actually use the urban landscape. Individuals which were observed only flying or singing were recorded for inventory purposes only.

### Response variables

We used two taxonomic variables—species abundance (S_Abund_) and richness (S_Rich_)–and two functional diversity indices—Rao’s Quadratic Entropy (RaoQ) and Functional Richness (FRic) as response variables. FRic represents the amount of functional space occupied by species in a community [20]. It is independent of species abundance, being a representation of functional trait space filled by the community [30]. Low functional richness indicates that some of the resources potentially available to the community are unused [21]. RaoQ incorporates species relative abundances and pairwise functional differences between species [31]. This index is an indirect measure of functional evenness, since the higher the value of RaoQ, the greater the dissimilarity between species, hence high functional evenness. To calculate the functional diversity indices aquatic species (we registered only one individual of great egret *Ardea alba*) and species that were not fully identified were not considered.

To calculate the functional diversity indices, we constructed a matrix containing two continuous traits and three categorical (fuzzy) traits (Table 1). The selected functional traits represent bird phenotypic characteristics which are influenced by environmental changes [32]. Data for the functional traits were collected from published literature [28,33,34].

**Table 1:** Functional traits used to calculate the functional diversity indices for the bird community inhabiting the streets of the southern region of Belo Horizonte, Minas Gerais, Brazil.

Trait category	Trait	Type of variable
**Morphological**	Body mass	Continuous
**Reproductive effort**	Clutch size	Continuous
**Nesting substrate**	Tree nester	Categorical
	Shrub nester	
	Primary excavator	
	Secondary excavator	
	Ground nester	
	Brood parasite	
**Foraging substrate**	Ground	Categorical
	Upper/medium foliage	
	Lower foliage	
	Air	
**Diet**	Mammals	Categorical
	Amphibians	
	Birds	
	Carrion	
	Invertebrates	
	Fruits	
	Seeds	
	Flowers/néctar	

Prior to calculating the functional diversity indices, we converted the fuzzy variables to proportional variables [35]. This was done using the prep.fuzzy function of the R [36] package ade4 [37]. Subsequently, the trait matrix was converted to a distance matrix using the dist.ktab function. This final matrix was used to calculate the functional diversity indices in R using the function dbFD of the FD package [38].

### Predictor variables

The information about human impacts and street trees and landscape characteristics were extracted from a buffer defined around the 60 point counts (Fig 2). The buffer radius definition aimed to find a balance between the size of the area and the amount of information about the urban landscape. Thus, we used the average variety function of the zonal statistics tool of ArcGIS 10.4.1 software to evaluate the amount of information of the land use/ land cover map [26] within buffers of different sizes (50 to 180m radius). We observed that 140m radius buffer size was the most informative (Fig 2, S2 Fig). Therefore, instead of using the 50m radius used to collect information about the urban bird community, we decided to use the 140m radius buffer to extract the following eight predictor variables (Table 2), since it provided more information about this urban landscape. For more information about the predictor variables definition see S2 Text.

**Table 2:** Predictor variables selected to assess the influences of human impacts and urban vegetation on taxonomic data and functional diversity indices of the bird community inhabiting the streets of Belo Horizonte (Minas Gerais, Brazil).

Variable category	Variable
Human impacts	Exposure to noise
	Human population
Trees characteristics	Proportion of the abundance of native street tree species
	Steet tree species richness
	Average diameter of street trees canopy
Landscape characteristics	Average arboreal patch size
	Average herbaceous patch size
	Average distance to parks

The human impact related variables were exposure to noise and the number of inhabitants (Table 2). A decibel meter (model Instrutherm DEC-490) was used to measure the sound pressure level at each point count location simultaneously with the bird sampling data. The decibel meter was calibrated (model Instrutherm CAL-4000) every day before data collection. An Equivalent Continuous Sound Level (L_eq_) index was calculated each sampling day for each point count, and the exposure to noise was measured through the calculation of the average L_eq_. This index is a measure of the overall level of exposure to sound in the environment. The human population (H_pop_) inside each buffer was estimated through data from the population census of the Brazilian Institute of Geography and Statistics from the year 2010 [39]. The proportion of people living inside of the area of the census sectors that overlapped the area of each buffer was calculated (Fig 2).

The landscape characteristics were the average arboreal patch size (Arb_patch_), the average herbaceous patch size (Herb_patch_), and the average distance to parks (Dist_parks_) (Table 2). Vegetation patches were comprised by clusters of pixels that were composed of arboreal or herbaceous vegetation in the land use/land cover map of the study area [26] (Fig 2). The average distance to parks for each point count was calculated using average Euclidian distance from all urban parks located within the study area (Fig 2).

The characteristics of street trees were the average diameter of the trees’ canopies (T_canopy_), tree species richness, (T_rich_), and the proportion of the abundance of native tree species (T_native_) (Table 2). This information was acquired from Belo Horizonte’s Tree Information System (SIIA-BH), which was designed to register information on street trees to promote the creation of a management tool [40]. Belo Horizonte municipal government consider these trees to be part of its ecological, landscape and cultural patrimony [40]. Approximately 90,000 street trees of 475 species were inventoried and georeferenced through the southern region of Belo Horizonte. We extracted only the street trees that were located within the 140m radius buffers around the point counts (Fig 2).

### Statistical analysis

We generated Generalized Linear Models (GLMs) between the response variables (species abundance, species richness, FRic and RaoQ) and the predictor variables. First, we performed a sensitivity analysis through the src function in R—a sensitivity package—to identify the influence of each predictor variable on the response variables; those predictor variables explaining less than 5% of variation were excluded from following analyses (S3 Fig). Therefore, each response variable has a distinct set of candidate models, composed of the most relevant combinations of predictor variables (S1 Table, S3 Fig). Most models were univariate, but we also compared multivariate additive models combining human impacts with landscape and street trees characteristics. To verify if the models were better than would be expected by chance, we included a null model representing the absence of effect of predictor variables. For the functional diversity indices, GLMs were generated with Gaussian distribution. For species richness and species abundance, Poisson distribution was used. Average arboreal patch size, average herbaceous patch size and human population were log-transformed.

We used a competing model selection approach to select the most plausible models [41]. For each model, we calculated Akaike’s information criterion for small sample sizes (AICc), and the difference in AICc between each model and the model with the lowest AICc (ΔAICc). Models with ΔAICc < 2.0 were considered to have substantial support [41], and then equally plausible. Furthermore, predicted relationships between response variables and the predictor variables included in the models with the lowest AICc value were plotted. Considering all combinations, we generated 15 candidate models for species richness, 8 models for FRic and 12 models for species abundance and RaoQ (S1 Table). Statistical analyses were carried out using the R packages AICcmodavg, MuMin, sensitivity and vegan [42–44].

## Results

Seventy three bird species were registered (almost 20% of all species registered in Belo Horizonte territory, which includes wetlands and natural habitat patches such as Atlantic Forest and Cerrado remnants—[45]), distributed in 26 families and 12 orders (Table 3). The most diverse family in terms of number of species was Tyrannidae, which represented almost 30% of the observed species (Table 3). The number of species registered in each point count location varied between 1 to 25 ($\bar{x}$ = 12.75, sd±4.47); 3143 individuals were counted, and varied from 2 to 143 per point count ($\bar{x}$ = 52.38, sd±30.93). The point count with a single species was excluded from our analysis, as it was not possible to calculate the functional diversity indices.

**Table 3:** Bird species observed resting or performing behaviors (such as nesting or feeding), and the total number of individuals (abundance) and number of point counts in which they were observed through the streets of the southern region of Belo Horizonte (Minas Gerais, Brazil). Scientific names and taxonomic order according to Piacentini et al. [46].

Order	Family	Species	Frequency	Abundance
Pelecaniformes	Ardeidae	*Ardea alba*	1	1
Cathartiformes	Cathartidae	*Coragyps atratus*	1	1
Accipitriformes	Accipitridae	*Rupornis magnirostris*	1	2
Columbiformes	Columbidae	Columbina talpacoti*^#^	45	423
		Columba livia*	30	357
		*Patagioenas picazuro*^#^	46	216
		*Patagioenas cayennensis*	1	4
Cuculiformes	Cuculidae	*Piaya cayana*	12	18
		*Crotophaga ani*	1	2
Strigiformes	Strigidae	*Athene cunicularia*	1	1
Apodiformes	Trochilidae	*Eupetomena macroura*	43	93
		*Anthracothorax nigricollis*	1	1
		*Chlorostilbon lucidus*	1	1
		*Amazilia lactea*	13	21
		Trochilidae sp.	2	2
Piciformes	Picidae	*Picumnus cirratus*	3	3
		*Colaptes campestris*	1	1
		*Colaptes melanochloros*	2	2
Falconiformes	Falconidae	*Caracara plancus*	4	5
		*Milvago chimachima*	4	6
		*Falco sparverius*	2	2
Psittaciformes	Psittacidae	*Psittacara leucophthalmus*	6	37
		*Forpus xanthopterygius*	6	33
		*Brotogeris chiriri*	14	152
Passeriformes	Thamnophilidae	*Thamnophilus caerulescens*	1	1
	Furnariidae	*Furnarius rufus*	16	43
	Tyrannidae	*Camptostoma obsoletum*	1	4
		*Elaenia flavogaster*	9	25
		*Serpophaga subcristata*	3	10
		*Myiarchus ferox*	2	3
		*Myiarchus tyrannulus*	3	4
		*Pitangus sulphuratus*^#^	46	193
		*Machetornis rixosa*	10	16
		*Myiodynastes maculatus*	2	2
		*Megarynchus pitangua*	10	21
		*Myiozetetes similis*	30	72
		*Tyrannus melancholicus*^#^	53	251
		*Tyrannus savana*	4	6
		*Empidonomus varius*	25	57
		*Colonia colonus*	2	4
		*Myiophobus fasciatus*	1	1
		*Fluvicola nengeta*	7	11
		*Lathrotriccus euleri*	1	1
		*Knipolegus lophotes*	1	1
		*Satrapa icterophrys*	1	1
		*Xolmis cinereus*	4	6
	Hirundinidae	*Pygochelidon cyanoleuca*	3	15
		*Progne tapera*	1	3
	Troglodytidae	*Troglodytes musculus*	10	18
	Turdidae	*Turdus leucomelas*	26	62
		*Turdus rufiventris*	2	2
		*Turdus amaurochalinus*	29	54
	Mimidae	*Mimus saturninus*	16	51
	Passerellidae	*Zonotrichia capensis*	2	7
	Parulidae	*Geothlypis aequinoctialis*	1	2
		*Basileuterus culicivorus*	2	4
	Icteridae	*Chrysomus ruficapillus*	1	10
		*Molothrus bonariensis*	19	33
	Thraupidae	*Tangara sayaca*	41	125
		*Tangara palmarum*	12	25
		*Tangara ornata*	1	2
		*Tangara cayana*	14	37
		*Sicalis flaveola*	8	39
		*Hemithraupis ruficapilla*	1	2
		*Volatinia jacarina*	6	23
		*Dacnis cayana*	2	4
		*Coereba flaveola*	42	142
		*Sporophila collaris*	1	1
		*Sporophila nigricolis*	1	3
		*Sporophila* sp.	2	5
	Fringillidae	*Euphonia chlorotica*	5	12
	Estrildidae	*Estrilda astrild*	8	76
	Passeridae	Passer domesticus*	39	268

* Most abundant species

^#^ Species most frequently observed throughout the point count locations

The most abundant species was the ruddy ground-dove *Columbina talpacoti* (423 individuals; 13% of all individuals), followed by two exotic species, the rock dove *Columba livia* (357 individuals; 11%) and the house sparrow *Passer domesticus* (268 individuals; 8.5%) (Table 3). Summed they represented 33% of all registered individuals. Most species (64 species, 87%) were represented by less than 100 individuals (Table 3). The tropical kingbird *Tyrannus melancholicus* was the most widely observed species in 88% of the point counts, followed by the Picazuro pigeon *Patagioenas picazuro*, the great kiskadee *Pitangus sulphuratus* and the ruddy ground-dove, which were recorded at c.a. 75% of the point counts (Table 3). Most species were observed in few point counts, there were 42 species (58%) registered in less than five point counts (Table 3).

**Fig 3:**
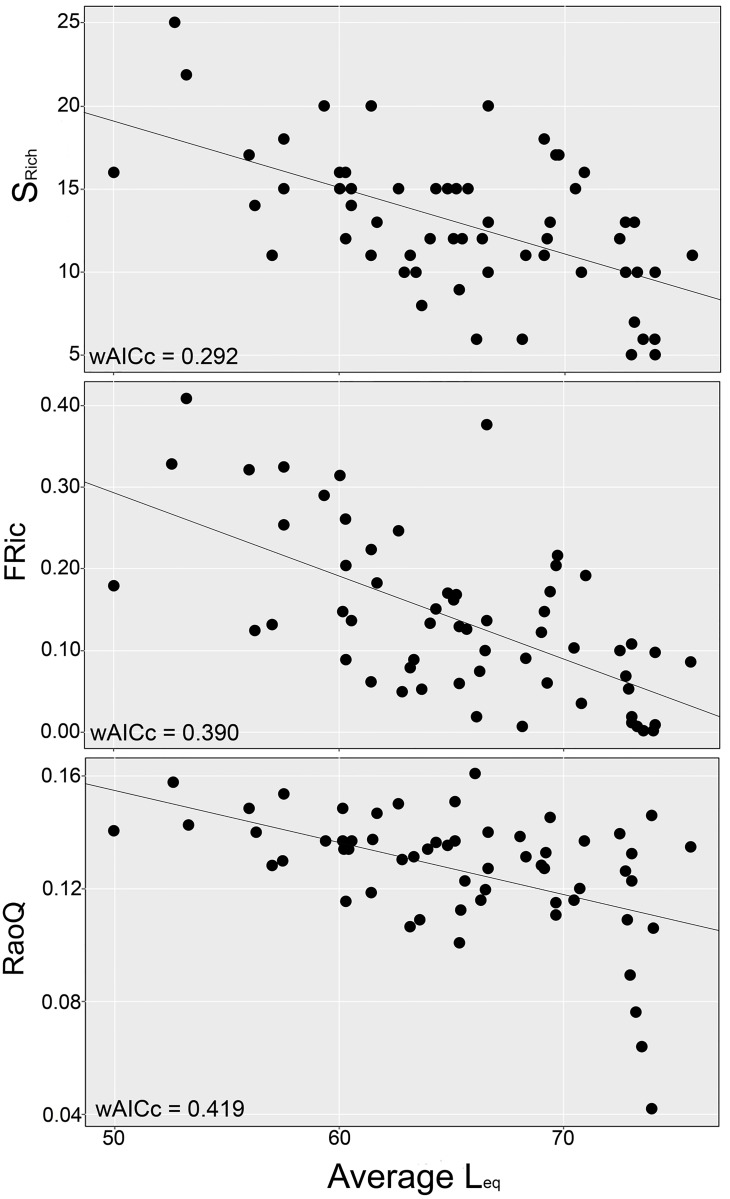
Best ranked univariate models, showing the negative influence of the exposure to noise (Average Equivalent Continuous Sound Level—L_eq_) on Species Richness (S_Rich_), Functional Richness (FRic) and Rao’s Quadratic Index (RaoQ), of the bird community inhabiting the streets of Belo Horizonte (Minas Gerais, Brazil).

**Fig 4:**
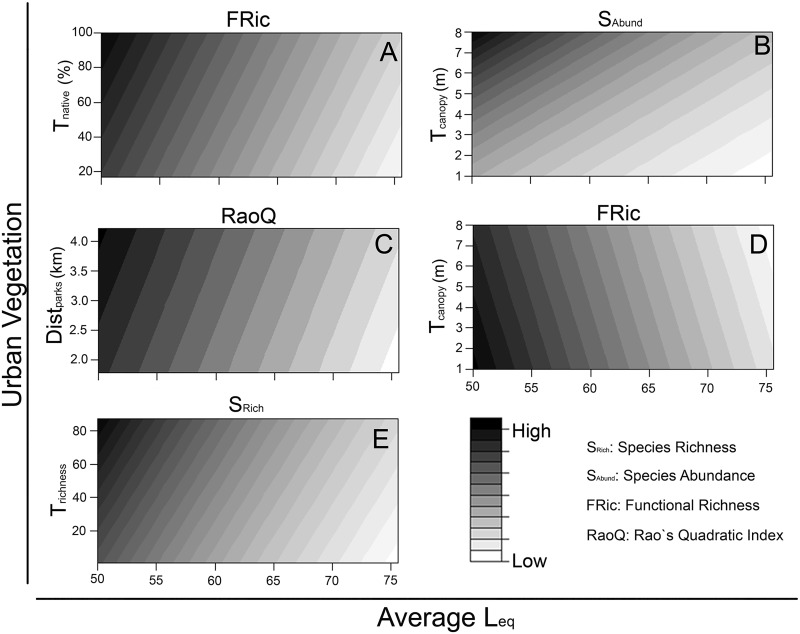
Patterns obtained in the multivariate models assessing the effects of the exposure to noise (Average Equivalent Continuous Sound Level—L_eq_) and urban vegetation variables on the taxonomic data and functional diversity indices of the bird community inhabiting the streets of Belo Horizonte (Minas Gerais, Brazil). T_native_: proportion of the abundance of native street tree species; T_rich_: street tree species richness; T_canopy_: average diameter of street trees canopy; Dist_parks_: average distance to parks.

RaoQ and FRic index scores were generally low, varying from 0.042 to 0.161 and from 0.001 to 0.409, respectively. Exposure to noise was included in all the best ranked models (Table 4), negatively affecting species richness, species abundance, FRic and RaoQ (Table 4, Figs 3 and 4). The univariate model containing exposure to noise was the most plausible model for species richness (wAICc = 0.292), FRic (wAICc = 0.390) and RaoQ (wAICc = 0.419) (Table 4, Fig 3). Five different patterns were found in the multivariate models with the exposure to noise (Fig 4). In general, the street trees and landscape characteristics had positive effects on the response variables, even with the negative effects of the exposure to noise (Table 4, Fig 4). However, average distance to parks had positive effects on RaoQ (Fig 4C) and the average size of the canopy of street trees had negative effects on FRic (Fig 4D), contradicting our expectations. The remaining models of FRic, RaoQ and species richness presented similar patterns to the ones shown in Fig 4A, 4C and 4E, respectively (S4 Fig). Human population and the average herbaceous patch size did not contribute to explaining patterns in the response variables (S1 Table).

**Table 4:** Best ranked models (AICc > 2.0) showing the influences of the exposure to noise (Average Equivalent Continuous Sound Level—L_eq_) and vegetation variables on taxonomic data and functional diversity indices of the urban bird community inhabiting the streets of the southern region of Belo Horizonte, Minas Gerais, Brazil.

Response Variable	Model	ΔAICc	wAICc	Slope sign
**S**_**Ric**_	~L_eq_	0	0.292	-
	~L_eq_ + T_native_	0.158	0.270	-
	~L_eq_ + Arb_patch_	1.034	0.174	-
	~L_eq_ + T_rich_	1.211	0.159	-
**S**_**Abund**_	~L_eq_ + T_canopy_	0	1	-
**FRic**	~L_eq_	0	0.390	-
	~L_eq_ + Arb_patch_	0.835	0.257	-
	~L_eq_ + T_native_	1.458	0.188	-
	~L_eq_ + T_canopy_	1.732	0.164	+
**RaoQ**	~L_eq_	0	0.419	-
	~L_eq_ + Dist_parks_	1.350	0.213	-
	~L_eq_ + Arb_patch_	1.422	0.206	-
	~L_eq_ + T_canopy_	1.932	0.160	-

S_Rich_: species richness; S_Abund_: species abundance; FRic: functional richness; RaoQ: Rao’s Quadratic Entropy; T_native_: proportion of the abundance of native street tree species; T_rich_: street tree species richness; T_canopy_: the average diameter of street tree canopy; Arb_patch_: average arboreal patch size; Dist_parks_: average distance to parks.

## Discussion

Street trees reduced the negative effects of the exposure to noise on the urban bird community inhabiting the streets of a Neotropical city. Urban areas with higher proportion of native street tree species and higher street tree species richness had a greater number of bird species and higher functional richness, even with the negative effects of the exposure to noise. Regions with larger trees had a greater number of birds across all species and less dominance of functional traits. Therefore, the urban bird community was more influenced by characteristics of street trees than characteristics of the landscape.

The lack of influence of the average herbaceous patch size is probably related to the reduced amount of native open habitat patches through this landscape. The majority of herbaceous vegetation patches were composed of cultivated lawns, squares and gardens, and utilized by open habitat generalist species such as the shiny cowbird (*Molothrus bonariensis*), the cattle tyrant (*Machetornis rixosa*) and the saffron finch (*Sicalis flaveola*). These species are commonly found in Belo Horizonte and other urban landscapes in southeastern Brazil [28,29]. Moreover, human population was not significant in any of the most plausible models, indicating that even in highly populated and dynamic urban centers it is possible to find a considerable amount of native biodiversity.

### Effects on species and taxonomic variables

On the streets of the southern region of Belo Horizonte it was possible to find 20% of all bird species recorded in the city’s territory [45]. To persist within the urban matrix, bird species need to adapt to an intense interaction with humans and different environmental impacts [7]. Therefore, only few species—defined as “urban exploiters” and “suburban adapters” [6]—will be able to exploit the reduced amount of resources and stressful conditions of streets. The environmental filter caused by the urbanization process [47,48], makes the most sensitive species locally extinct and generalist species, especially omnivorous species become dominant [49]. However, bird communities have different responses to urbanization worldwide. While in temperate urban landscapes urban bird communities are composed mostly of omnivorous and seed eaters [7,49–52], in Neotropical urban centers (as observed in Belo Horizonte), omnivorous and insectivorous species are dominant [53–56]. Urban bird communities are shaped by the availability of resources [49]. Unlike temperate urban landscapes, warmer temperatures of most Neotropical cities probably allow for larger populations of insects and the presence of a larger number of insectivirous birds species. Different groups of arthropods are abundant in urban landscapes, such as generalist ground arthropods, plant-feeding arthropods and generalist pollinating arthropods [57]. Domestic wastes also provide opportunities for insectivorous birds to feed on insects such as flies and mosquitoes [58]. Furthermore, the high diversity of tyrant flycatchers (Tyrannidae) in Neotropical cities [53–56,59], probably have a positive effect on the larger number of insectivorous species that occupy Neotropical urban landscapes.

The negative effects of the exposure to noise on urban birds has been evaluated in many studies [14,15,59]. However, our study is the first to assess, at the landscape level, how the exposure to noise influences the bird community occurring in the streets of an urban landscape. Noise intensity can be considered as a proxy for other negative effects within streets: the higher the exposure to noise, the greater the impacts associated with urbanization, and therefore, the smaller the number and abundance of bird species able to occupy streets. Our results show that, in addition to helping species conservation within protected areas [9,60], the appropriate planning and management of the urban afforestation process—such as increase the number of large and native tree species in the streets—is able to mitigate the negative effects of the urbanization on birds that occupy the urban matrix. Urban bird species richness is positively influenced by the amount of native street tree species [9,18,53,61,62]. This is related to birds’ preferences for native tree species as nesting sites [61] and the availability of resources, such as arthropods, which is higher in native trees [63]. Larger trees increase canopy complexity and provide critical resources for the native fauna inhabiting urban landscapes, such as a large contribution to flower, fruit and seed production and provision of cavities [64]. Therefore, large trees have a strong positive effect on different taxa, increasing species abundance and richness, and are considered keystone ecological structures in urban landscapes [62,64–66]. Furthermore, trees also can act as sound barriers causing sound to disperse and dissipate [67].

The reduced influence of the distance to urban parks on bird species richness and number of individuals is probably related to the high efficiency of the local avifauna in exploring this urban landscape—the most frequent species in our landscape were native bird species, such as the ruddy ground-dove, the great kiskadee and the Picazuro pigeon. Bird species inhabiting streets may efficiently move through the landscape, perceiving the urban matrix as a *continuum* of habitats, and are influenced by local conditions such as the characteristics of street trees [11]. However, species composition change with the distance to urban parks, and this is confirmed by the functional diversity results.

### Effects on functional diversity indices

Community’s functional aspects presented low values across the streets of the southern region of Belo Horizonte, which corroborates the results for the taxonomic richness. Probably, the resources available across the landscape are low [21], filtering species that have specific ecological requirements. Most bird species found in this study are omnivorous, feeding on invertebrates and/or using human-made structures as nesting substrates, leading to a reduction in the community’s functional richness. This result is confirmed through the low RaoQ index scores, showing that species with similar functional traits are dominant. The urbanization process leads to a reduction in the amount of bird species’ traits, particularly in traits related to resource use and nesting substrate [52,68]. However, our results are in accordance with another study, in which the increase in the amount of arboreal vegetation led to an increase in the community’s functional aspects [69]. The increase in the proportion of native street tree species influence the presence of a larger amount of bird feeding guilds in urban landscapes [9,18], and consequently had positive influences on community’s functional richness.

Urban landscapes have large quantities of resources for a portion of the avifauna with similar functional traits [52,68]—which is related to the general low functional richness and RaoQ index scores. Since synanthropic species are negatively influenced by the amount of habitat within urban landscapes [8], urban areas with more available habitat will allow the presence of species with functional traits related to the most preserved areas, leading to changes in the functional diversity indices. The average canopy size of the street trees increases community’s RaoQ and leads to a reduction in the community’s functional richness. Probably, the increase in canopy size reduces the richness and abundance of a larger number of species that have functional traits related to more urbanized areas; the increase in number of birds, probably, is related to an increase in the abundance of fewer species, which have functional traits related to more preserved areas. This process increases community’s functional evenness with a lower number of functional traits. The proximity to parks can reduce the total number of individuals for many bird species, which have functional characteristics related to the more urbanized areas [25,68]. However, species that need a greater amount of habitat, probably, are favored, reducing taxonomic evenness without changing the taxonomic and functional richness, consequently reducing RaoQ. Therefore, despite the bird community inhabiting streets being composed mostly of species adapted to the conditions related to the urbanization process, it is possible to increase the number of species with functional traits related to most preserved environments.

## Conclusions

We demonstrated that the urban environmental filter has negative effects on urban avifauna. However, human impacts can be mitigated by the appropriate planning and management of urban vegetation, especially related to the urban afforestation process. The maintenance of large trees, increasing street tree richness and planting of a larger amount of native tree species are management practices able to increase urban biodiversity and ecosystem functionality within the urban matrix.

“We need nature as much in the city as in the countryside” was written by Ian McHarg in 1969 in his book *Design with nature* [70]. Fifty years later, we still need to learn how to enhance, preserve and live with biodiversity within urban landscapes. With the current planning and management practices, Belo Horizonte is only able to retain 20% of its rich and diverse bird community within the urban matrix. Therefore, we need to change the current focus on a purely aesthetic and utilitarian view of the urban afforestation process [71]; decisions must consider the functionality of the urban landscape and the green elements as interconnected units [72,73]. Considering that bird species can be used as indicators of urban ecological integrity [56], planning and management practices, especially those related to street trees identified here and in other studies [e.g.: 9,11,18,50,51,57,63] are able to reduce the negative effects of urbanization on biodiversity and consequently enhance human wellbeing and quality of life.

## Supporting information

S1 Fig The 60 point counts selected within 20 categories of influences of herbaceous and arboreal vegetation and streets through the southern region of Belo Horizonte (Minas Gerais, Brazil). (TIF)

S2 Fig Variation of the average variety of land use types of the southern region of Belo Horizonte (Minas Gerais, Brazil) within buffers with different sizes. (TIF)

S3 Fig Sensitivity analysis performed for species richness, species abundance, Functional Richness (FRic) and Rao’s Quadratic Entropy (RaoQ) of the bird community inhabiting the streets of the southern region of Belo Horizonte. Only predictor variables located outside the range of 0.05 and -0.05 (black lines) were considered. It is possible to observe the large effect of the exposure to noise on the taxonomic and functional aspects of the urban bird community. AP: average arboreal patch size; HP: average herbaceous patch size; DC: average size of the canopy of street trees; AL: average Equivalent Continuous Sound Level; TR: street tree richness; DP: average distance to parks; PT: human population; NT: proportion of the abundance of native tree species in the streets.
(TIF)

S4 Fig Multivariate additive models showing the influences of urban environmental variables on taxonomic data and functional diversity indices of the bird community inhabiting the streets of the southern region of Belo Horizonte (Minas Gerais, Brazil). S_Ric_: Species richness; S_Abund_: Species abundance; FRic: Functional richness; RaoQ: Rao’s Quadratic Index; L_eq_: Average Equivalent Continuous Sound Level; T_native_: proportion of the abundance of native street tree species; T_rich_: street tree species richness; T_canopy_: the average diameter of street tree canopy; Arb_patch_: the average arboreal patch size; Dist_parks_: the average distance to parks.
(TIF)

S1 Table Candidate models defined for the response variables related to the bird community inhabiting the streets of the southern region of Belo Horizonte (Minas Gerais, Brazil), highlighting models with ΔAICc < 2.0. (PDF)

S1 Text Point counts’ selection. (PDF)

S2 Text Definition of predictor variables. (PDF)
