# Supplementary figures and images for: Street trees reduce the negative effects of urbanization on birds
Source: PLoS One. 2017 Mar 23;12(3):e0174484. doi: 10.1371/journal.pone.0174484 (PMC5363989; doi:10.1371/journal.pone.0174484)

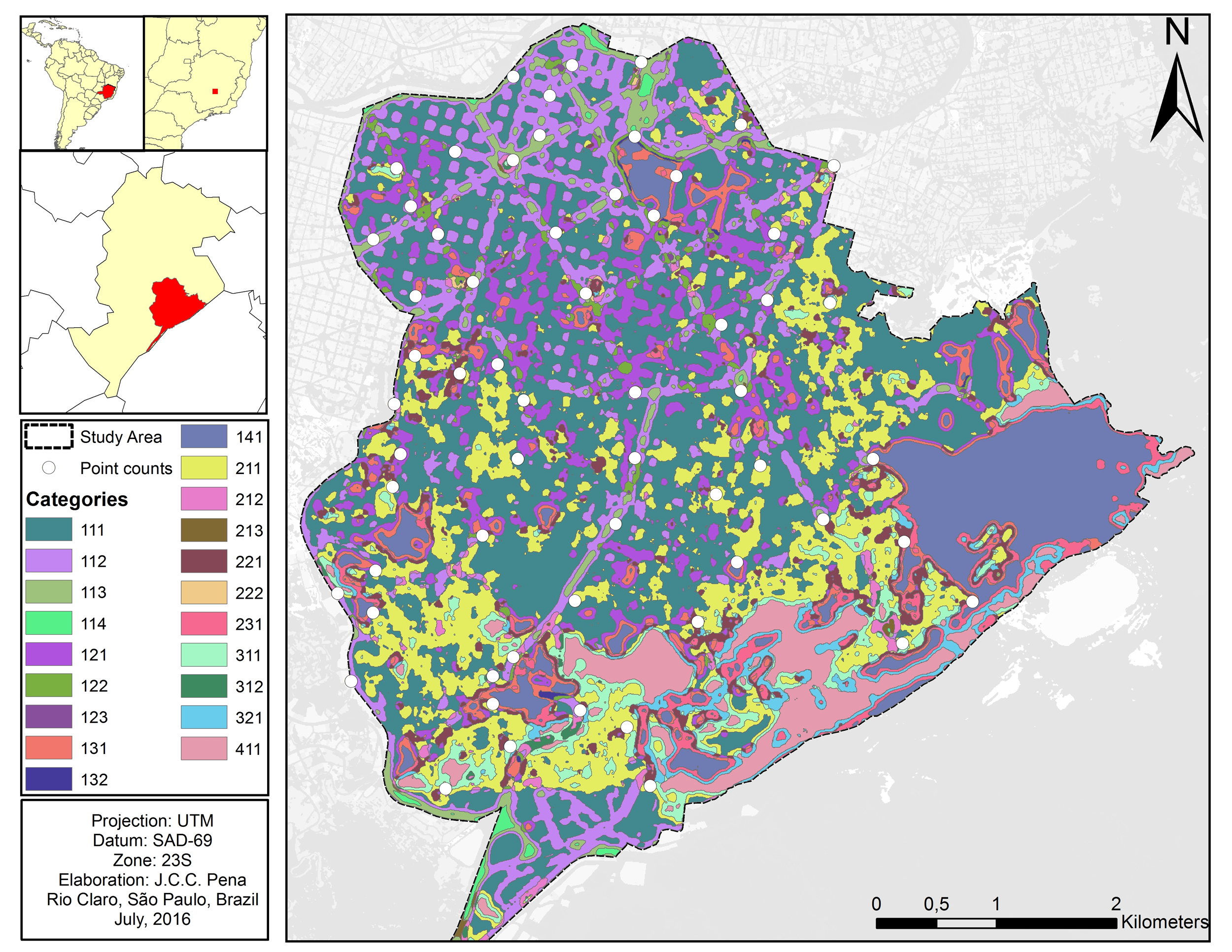

Supplement: S1 Fig — (TIF) [file pone.0174484.s001.tif]

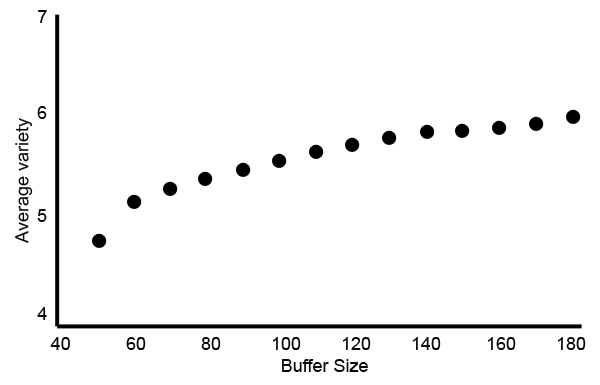

Supplement: S2 Fig — (TIF) [file pone.0174484.s002.tif]

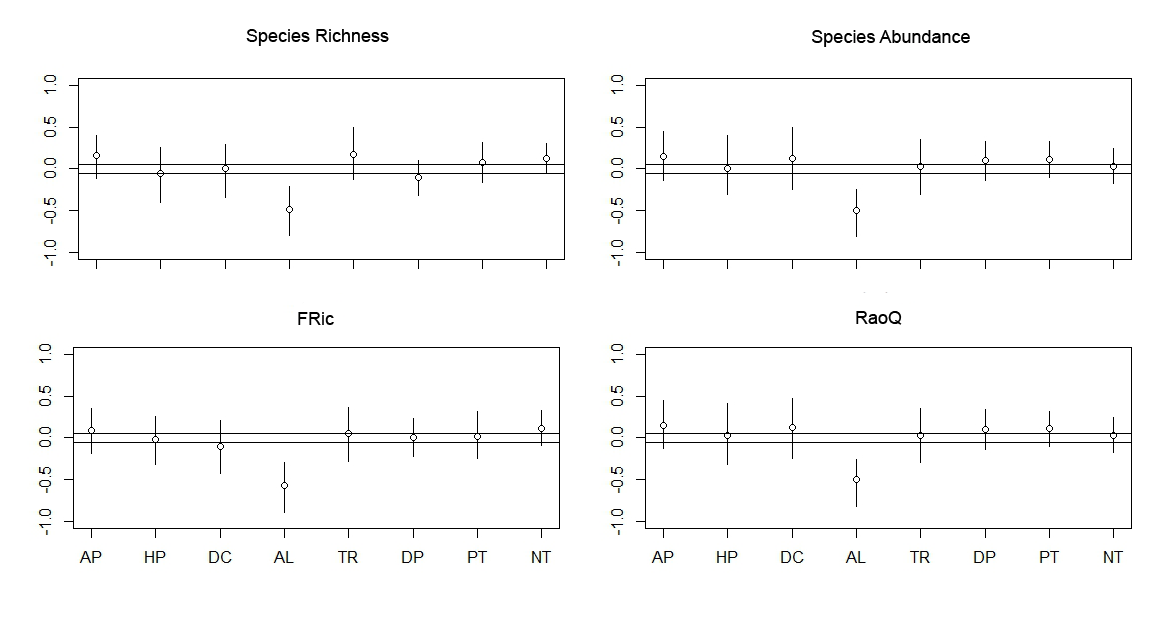

Supplement: S3 Fig — AP: average arboreal patch size; HP: average herbaceous patch size; DC: average size of the canopy of street trees; AL: average Equivalent Continuous Sound Level; TR: street tree richness; DP: average distance to parks; PT: human population; NT: proportion of the abundance of native tree species in the streets. (TIF) [file pone.0174484.s003.tif]

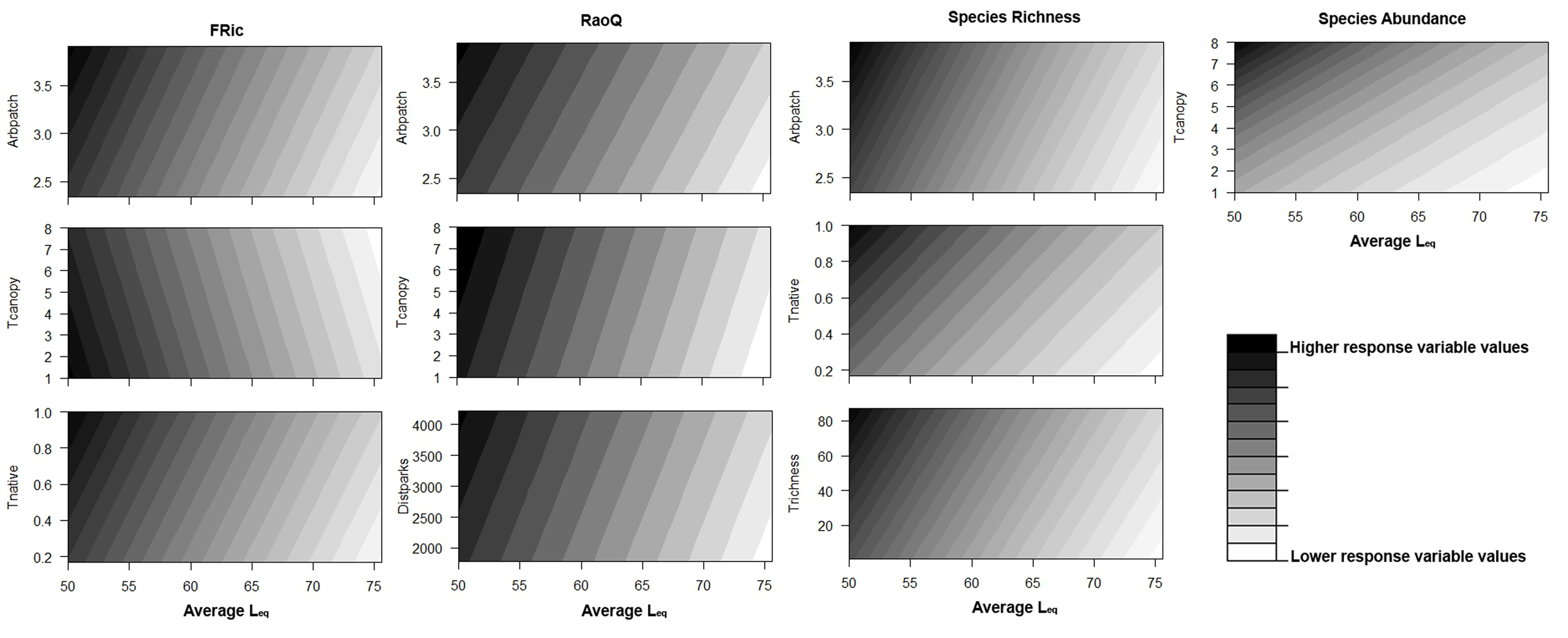

Supplement: S4 Fig — SRic: Species richness; SAbund: Species abundance; FRic: Functional richness; RaoQ: Rao’s Quadratic Index; Leq: Average Equivalent Continuous Sound Level; Tnative: proportion of the abundance of native street tree species; Trich: street tree species richness; Tcanopy: the average diameter of street tree canopy; Arbpatch: the average arboreal patch size; Distparks: the average distance to parks. (TIF) [file pone.0174484.s004.tif]
