# Supplementary material for: Street trees reduce the negative effects of urbanization on birds
Source: PLoS One. 2017 Mar 23;12(3):e0174484. doi: 10.1371/journal.pone.0174484 (PMC5363989; doi:10.1371/journal.pone.0174484)
Supplement: S1 Table — (PDF) [file pone.0174484.s005.pdf]

SUPPLEMENTARY MATERIAL

Street trees reduce the negative effects of urbanization on birds

S1 Table: Candidate models defined for the response variables related to the bird community inhabiting the streets of the southern region of Belo Horizonte (Minas Gerais, Brazil), highlighting models with ΔAICc < 2.0.

Response variable	Candidate Model	k	ΔAICc	wAICc
S_{Rich}	**$\sim L_{eq}$**	**2**	**0.000**	**0.292**
	**$\sim L_{eq} + T_{native}$**	**3**	**0.158**	**0.269**
	**$\sim L_{eq} + Arb_{patch}$**	**3**	**1.034**	**0.174**
	**$\sim L_{eq} + T_{rich}$**	**3**	**1.211**	**0.159**
	$\sim L_{eq} + Dist_{parks}$	3	2.047	0.105
	$\sim T_{rich}$	2	14.226	< 0.001
	$\sim H_{pop} + T_{rich}$	3	16.303	< 0.001
	$\sim Arb_{patch}$	2	22.774	< 0.001
	$\sim T_{native}$	2	22.874	< 0.001
	~null	1	23.153	< 0.001
	$\sim Dist_{parks}$	2	24.232	< 0.001
	$\sim H_{pop} + Arb_{patch}$	3	24.764	< 0.001
	$\sim H_{pop} + T_{native}$	3	24.979	< 0.001
	$\sim H_{pop}$	2	25.287	< 0.001
	$\sim H_{pop} + Dist_{parks}$	3	26.453	< 0.001
S_{Abund}	**$\sim L_{eq} + T_{canopy}$**	**3**	**0.000**	**1.000**
	$\sim L_{eq} + Dist_{parks}$	3	35.421	< 0.001
	$\sim L_{eq} + Arb_{patch}$	3	68.819	< 0.001
	$\sim L_{eq}$	2	68.919	< 0.001
	$\sim H_{pop} + Dist_{parks}$	3	98.160	< 0.001
	$\sim Dist_{parks}$	2	101.464	< 0.001
	$\sim H_{pop} + T_{canopy}$	3	129.930	< 0.001
	$\sim T_{canopy}$	2	132.318	< 0.001
	~null	1	148.887	< 0.001
	$\sim H_{pop}$	2	149.107	< 0.001
	$\sim H_{pop} + Arb_{patch}$	3	150.546	< 0.001
	$\sim Arb_{patch}$	2	150.739	< 0.001
FRic	**$\sim L_{eq}$**	**3**	**0.000**	**0.390**
	**$\sim L_{eq} + Arb_{patch}$**	**4**	**0.835**	**0.257**
	**$\sim L_{eq} + T_{native}$**	**4**	**1.458**	**0.188**
	**$\sim L_{eq} + T_{canopy}$**	**4**	**1.732**	**0.164**
	$\sim T_{canopy}$	3	23.735	< 0.001
	~null	2	26.731	< 0.001
	$\sim Arb_{patch}$	3	26.860	< 0.001
	$\sim T_{native}$	3	28.158	< 0.001
RaoQ	**$\sim L_{eq}$**	**3**	**0.000**	**0.419**

**$\sim L_{eq} + Dist_{parks}$**	**4**	**1.350**	**0.213**
**$\sim L_{eq} + Arb_{patch}$**	**4**	**1.422**	**0.206**
**$\sim L_{eq} + T_{canopy}$**	**4**	**1.932**	**0.160**
~null	2	13.059	0.112
$\sim Arb_{patch}$	3	13.825	0.076
$\sim T_{canopy}$	3	14.655	0.050
$\sim Dist_{parks}$	3	15.058	0.041
$\sim H_{pop}$	3	15.137	0.039
$\sim H_{pop} + Arb_{patch}$	4	15.660	0.031
$\sim H_{pop} + T_{canopy}$	4	15.687	< 0.001
$\sim H_{pop} + Dist_{parks}$	4	17.163	< 0.001
