# Supplementary material for: Street trees reduce the negative effects of urbanization on birds
Source: PLoS One. 2017 Mar 23;12(3):e0174484. doi: 10.1371/journal.pone.0174484 (PMC5363989; doi:10.1371/journal.pone.0174484)
Supplement: S1 Text — (PDF) [file pone.0174484.s006.pdf]

SUPPLEMENTARY MATERIAL

Street trees reduce the negative effects of urbanization on birds

S1 Text: Point counts' selection

To evaluate the influence of the urbanization on the bird community inhabiting the streets of the southern region of Belo Horizonte, the selection of sampling points aimed to consider the maximum possible interactions between urban features and the remaining arboreal and herbaceous vegetation. Thus, we adopted the methodology described below.

From the land use/land cover map [1] in raster format, we extracted, independently, the pixels containing information on built-up area, arboreal vegetation, herbaceous vegetation, and streets. In each of the four maps, the intensity in which each pixel is influenced by each variable was evaluated through a moving window with 50m radius (half of the size of a city block in Belo Horizonte – 100m). Thus, we obtained four maps classified in values between zero and one. Pixels with values closer to zero are related to a low influence of that land use type (indicating, for example, low influence of arboreal vegetation), and pixels with values closer to one are related to a high influence of that land use type (high influence of streets or build-up area, for example). The average intensity of the streets' map moving window is 0.29, arboreal map moving window is 0.40, herbaceous map moving window is 0.41 and build-up area map moving window is 0.54.

We then used the R software to assess the degree of correlation between streets and build-up area and the vegetation features (as the variables did not have a normal distribution, we used the Spearman coefficient). The built-up area map showed a high degree of correlation (0.64 $p < 0.05$) compared to the streets map (0.34 $p < 0.05$), therefore, it was excluded from our analysis. Thus, the three remaining moving window maps were reclassified to categorical values representing intensities of influence: streets 1 – 4; arboreal vegetation: 10 – 40; herbaceous vegetation: 100 – 400. Then the map algebra process was used to sum these three maps, and it was obtained a final map containing 20 categories of interactions between the three land use types (S1 Fig.).

In this final map were randomly created 10,000 points, from which we selected 3 points in each of the categories of land use interactions. These points were distributed throughout the streets of the study area and were at least 200 meters away from each other (S1 Fig.). The distribution of points between and in the same category aimed to achieve the greatest latitudinal and longitudinal range within the study area. Points located nearby or within risk areas (such as irregular occupations/slums) were not selected. The selected 60 point counts were distributed in regions with large amounts of arboreal and herbaceous vegetation but with low streets density, as well as within the city center, with high streets density, and small amount of herbaceous and arboreal vegetation (S1 Fig.). All spatial analyzes were carried out using Grass GIS 6.4 software.

References

1. Pena JCDC, Marques de Magalhães D, Clara Mourão Moura A, Young RJ, Rodrigues M. The Green Infrastructure of a Highly-Urbanized Neotropical City: the Role of the Urban Vegetation in Preserving Native Biodiversity. Rev da Soc Bras Arborização Urbana. 2016;11: 66–78.
