# Supplementary material for: Street trees reduce the negative effects of urbanization on birds
Source: PLoS One. 2017 Mar 23;12(3):e0174484. doi: 10.1371/journal.pone.0174484 (PMC5363989; doi:10.1371/journal.pone.0174484)
Supplement: S2 Text — (PDF) [file pone.0174484.s007.pdf]

SUPPLEMENTARY MATERIAL

Street trees reduce the negative effects of urbanization on birds

S2 Text: Definition of predictor variables

We selected 17 variables that we considered to be highly influential on the bird community inhabiting the streets: average distance to parks; human population size; average Equivalent Continuous Sound Level; average building height; proportion of vegetation within the buffer; the average arboreal patch size; the average herbaceous patch size; the average size of the canopy of the street trees; diversity of street trees (Shannon index); street tree richness; street tree abundance; the number of native street tree species; the proportion of native street tree species; the abundance of native street tree species; the proportion of the abundance of native street tree species; the abundance of fleshy fruit trees in the streets; the abundance of flowering trees in the streets.

A Principal Component Analysis (PCA) was performed to reduce the number of variables on principal component axes that retained as much variability as possible of our predictor variables. We retained the eight predictor variables described in the main text, that were highly correlated with the first four principal components. These four PCA axes explained 76% of landscape variance.
